# Emergency Preparedness in 2010 and Concerns About COVID-19: Evidence from the Health and Retirement Study

**DOI:** 10.1093/geroni/igab046.2683

**Published:** 2021-12-17

**Authors:** Timothy Killian, Betsy Garrison, Charleen McNeill

**Affiliations:** 1 University of Arkansas, Fayetteville, Arkansas, United States; 2 University of Arkansas, University of Arkansas, Arkansas, United States; 3 East Carolina University, East Carolina University, North Carolina, United States

## Abstract

Emergency preparations are particularly important for older persons as age-related vulnerabilities increase risk of morbidity and mortality associated with disasters. The novel COVID-19 virus combined with the ongoing efforts of the Health and Retirement Study (HRS) has provided researchers with the opportunity to examine how emergency preparedness was related to responses to the COVID-19 disaster approximately 10years later. The data for this study were generated from participants in the HRS.. This study only examined data from participants in both the disaster preparedness sub-sample of 2010 and COVID-19 sub-sample in 2020 resulting in data from 249 participants. In 2020, participants were asked how concerned they were about COVID-19 and whether or not they had been tested for the virus. This study found that disaster preparations in 2010 predicted lower levels of concern about COVID-19 in 2020. The differences in the means for all items, however, was not large enough to reach statistical significance with one exception, participating in an educational program about disaster preparedness (Wilcoxon-Z = -1.88, p < .05). Disaster preparations were associated with an increased likelihood of getting tested for COVID-19 for four of the six items. Statistical significance, however, was only achieved for a single item. Participants who had supplies for a three-day emergency in 2010 had higher rates of getting tested in 2020 (24.84%) as compared to those who did not have supplies for a three-day emergency (14.13%; χ2 = 4.03, p < .05). If accepted for presentation, implications for an array of audiences will be developed.

